# Omental Patch Technique for the Ileal Perforation Secondary to Typhoid Fever

**DOI:** 10.4103/1319-3767.80386

**Published:** 2011

**Authors:** Musharraf Husain, Rehan Nabi Khan, Babar Rehmani, Hasan Haris

**Affiliations:** Department of Surgery, J N Medical College, Aligarh, India

**Keywords:** Enteric perforation, omental patch, peritonitis

## Abstract

**Background/Aim:**

Enteric perforation is a grave complication of typhoid fever. Laparotomy with primary closure is the treatment of choice depending upon the bowel condition. Fecal fistula formation is the main concern in primary closure and the incidence of this complication dramatically decreases when omentum is used as a patch over primary closure.

**Materials and Methods:**

A total of 176 patients underwent laparotomy for enteric perforation and they were divided into two groups randomly; Group I–Primary closure with omental patch and GroupII– –Only primary closure. The outcomes were measured in relation to hospital stay, wound infection, septicemia, fecal fistula, and mortality.

**Results:**

The incidence of complications including fecal fistula and mortality is significantly lower in the group I patients. Fecal fistula occurs in 7.7% in group II, while in only 1.1% in group I. The mortality is also lower 3.3% in group II, while 1.1% in group I.

**Conclusion:**

Primary closure with omental patch is a better option as compared with only primary closure in enteric perforation patients. It can be recommended as an alternative method to primary closure only in enteric perforation patients.

Typhoid fever is a systemic disease characterized by fever and abdominal pain caused by dissemination of *Salmonella typhi*. This disease was initially called typhoid fever because of its clinical similarity to Typhus. In 1869, the term enteric fever was proposed as an alternative designation on the basis of its association with enlarged Peyer’s patches and mesenteric lymph nodes. There are a lot of complication of this disease if not treated properly and in time, but the two most dreaded complications related to surgeons are intestinal perforation and bleeding.[1] These complications result from necrosis at the initial site of *Salmonella* infiltration in the Peyer’s patches of the small intestine. This usually occurred in the third or fourth week of infection.[2]

Enteric perforation required immediate surgical intervention. Various treatment options are available as advocated by different authors depending on the bowel condition. The different procedures are—primary closure,[3] primary closure with omental patch,[4] resection and anastomosis,[5] ileostomy,[6] and primary closure with ileotransverse colostomy.[7]

The aim of our study is to compare the results of simple primary closure and primary closure with omental patch technique.

## MATERIALS AND METHODS

This study was carried out at a tertiary care referral hospital, during the period between June 2002 and February 2006. A total of 176 patients were included in the study, who were having enteric perforation peritonitis due to typhoid fever and qualified the criteria of primary closure, that is, single perforation with relatively healthy bowel.

Diagnosis of enteric perforation was made on the basis of prolonged fever followed by acute attack of abdominal pain with signs and symptoms of generalized perforation peritonitis. Widal test and blood culture were done in every patient. X-ray chest including both diaphragms were done as a routine investigation along with hemogram, blood sugar, blood urea, and serum creatinine.

All the patients were resuscitated with intravenous fluids. Nasogastric aspiration and urethral catheterization was done in every patient. Preoperative antibiotics, ceftriaxone (1 gm) and metronidazole (500 mg), were given. Exploratory laparotomy under general anesthesia was carried out within 24 hours of admission. The entire bowel was explored and its findings were noted in relation to amount and nature of fluid, site, size, and number of perforations and condition of the adjacent bowel. Surgical procedure was done according to the following criteria.

Primary closure—Single perforation with relatively healthy bowel

Ileostomy—Single or multiple perforation with unhealthy bowel and poor-risk patients

Resection and anastomosis—Multiple perforation with relatively healthy bowel

Only those patients who met the criteria of primary closure (single perforation with relatively healthy bowel) were included in the study. These patients were randomly distributed alternatively into two groups.

Group I - Primary closure with omental patch

Group II - Primary closure only

A total of 302 patients of enteric perforation were treated at the hospital during the above mentioned period. Of them, 176 patients underwent exploratory laparotomy with primary closure of perforation; ileostomy was done in 93 patients, resection anastomosis in 30 patients, while drains were put in three patients under local anesthesia. All the three patients with the drain expired and could not undergo definitive surgery. Only those 176 patients who had single perforation with relatively healthy bowel were included in the study.

### Technique

Primary closure was done in two layers with interrupted sutures. 3-0 vicryl was used for inner layer, while 3-0 silk was used for outer layer.

In first group, omentum was brought down and fixed at the perforation site with the help of 3-0 vicryl, like a patch after the primary closure.

Cavity was cleaned with the help of 2.5 l of normal saline before applying the patch. Single drain (30F) was put in the pelvis and abdominal closure was done in single layer. Postoperatively, the patients were given injections ceftriaxone and metronidazole for 5 days.

The patients were observed with respect to wound infection, wound dehiscence, septicemia, development of fecal fistula, and mortality.

## RESULTS

A total of 176 patients who underwent exploratory laparotomy with primary closure of perforation were included in the study. Of the 176, 90 patients underwent primary closure with omental patch (Group I), while 86 patients underwent primary closure of perforation only (GroupII).

Of 176 patients, 75 (43%) patients were in the age group of 21 to 30 years, 65 (37%) were in the age group of 10 to 20 years, while the remaining 36 patients were more than 30 years of age. Majority of the patients were males, 144 (82%). The median age in group I was 18.5, while the median age in group II was 19.2.

A total of 116 (66%) patients were presented within 48 hours of beginning of abdominal pain, while 60 (34%) were presented with 3- to 4-day old perforation. None of the patients were presented after 4 days in our selected group. 63.3% (57) of the patients were presented within 48 hours in group I, while 68.6% (58) of patients were presented within 48 hours in group II.

Classic history of fever followed by abdominal pain was present in 161 (91.4%) patients and only 15 patients presented with abdominal pain without any history of fever.

Pneumoperitoneum was detected in 163 (92.6%) patients, Widal test was positive in 98 (55.7%) patients, while blood culture was positive in 23 (13%) patients.

All patients were monitored for early postoperative obstruction and other complications, as shown in Table 1. Differences in the incidence of complications in two groups were assessed with the help of 2-sided Fischer’s exact test to know whether they are statistically significant or not. *P* value <0.05 is taken as significant.

**Table 1:** Post operative complications

Complications	Group 1 (90 patients)	Group 2 (86 patients)	*P* value
Wound infections	33 (36.6)	37 (41.1)	0.4421
Wound dehiscence	6 (6.6)	7 (7.7)	0.7785
Intrabdominal abscess	5 (5.5)	9 (10)	0.2729
Fecal fistula	1 (1.1)	7 (7.7)	0.0316
Septicemia	3 (3.3)	9 (10)	0.0758
Mortality	1 (1.1)	3 (3.3)	0.3595

Figures in parentheses are in percentage

Most common complication following the procedure was wound infection which was almost equal in both the groups. There is not much difference in the complication rates in both the groups, except the occurrence of fecal fistula which is also statistically significant. None of the patients developed postoperative obstruction because of omentum. Duration of hospital stay was ranging from 5 to 23 days.

## DISCUSSION

Enteric perforation is more common in the age group of 21 to 30 years in our study (43%) which is consistent with other studies, as reported in Singh *et al*. and Olurin *et al*.[6,8]

Majority of our patients were males (82%). Adensunkanni *et al*. and Swadia *et al*. reported male to female ratio of 4 : 1 and 5.25 : 1, respectively,[9,10] while Beniwal *et al*. showed a ratio of 6.4 : 1.[11] This higher incidence in young males is because they are more exposed to infections as they eat more outside and have poor hygiene.

Most of the studies showed delayed presentation after the acute abdominal pain. Mean duration of 5.6 days was reported by Naaya *et al*.,[12] while the range was 1 to 7 days in a series by Mansoor *et al*.[13] Most of our patient presented within 48 hours of abdominal pain, 116 (66%). This early presentation for the majority of the patients may not be the true representation because we have taken only selected patients for our study.

In most of the series, the patient presented with a history of 1 to 2 weeks of fever followed by pain abdomen.[11,14] Of all, 161 (90%) of our patients presented with symptoms of fever followed by pain.

The diagnosis of enteric perforation was made mainly on the basis of clinical history and examination, X-ray abdomen, and Widal test. Widal test was positive in only 98 (55.6%) of our patients, whereas Beniwal *et al*. showed a positivity of 80.5% in their series. Pneumoperitoneum could be detected in 163 (92.6%) patients, which is consistent with other reports like Beniwal *et al*. reporting a detection rate of 91.7%.[11]

Enteric fever is best managed by early surgical intervention. Various surgical options available are simple primary closure, primary closure with omental patch, resection and anastomosis, and closure with ileotransverse colostomy. All these different procedures were tried, to decrease the incidence of fistula formation and its associated morbidity and mortality. Fecal fistula may occur either because of reperforation or different site of perforation. Simple primary closure is still the procedure of choice as it is simple, quick, and cost-effective.[11] It also avoids stoma care and its complications. Omentum is used to reinforce the simple primary closure by few surgeons. We have compared the results of simple primary closure and primary closure with omental patch technique. Use of omentum is not new in surgery. It has been used almost in every subspecialty of surgery, more commonly in GI surgery. It may be used as Graham’s patch in peptic ulcer perforation, wrapping around an anastomosis, esophageal surgery, and others like omentoplasty in hydatid cyst. The omentum fills in small gaps between the sutures and provide good source of vascularity and inflammatory cells for healing. Due to its capacity of localizing infection and sealing microperforations, some of the authors strongly recommended omentoplasty in every case of anastomosis, especially the esophagus and colorectal.[15]

We have compared the results of two groups and found that leak rate is lower in patients with primary closure and omental patch. The occurrence of fistula formation is mainly after the primary closure of perforation. However, some of the studies showed that the development of fecal fistula and mortality is unrelated to the operative procedure.[13] Mansoor *et al*. showed that maximum incidence of fecal fistula is with resection and anastomosis followed by simple repair closure (7%) and no fistula formation in ileostomy group. Mortality is minimal with simple primary closure, as shown by Mansoor *et al*, Talwar *et al*, and Beniwal *et al*. showed no difference in the mortality among different surgical groups and the mortality depends on number of perforations and development of fecal fistula.[11,13,14] The reason of low mortality in primary repair groups in the above studies may be because of early presentation and relatively healthy bowel of these patients. The incidence of fecal fistula as shown in different studies is 16.6% according to Olurin *et al*., 10% according to Talwar *et al*., 8.5% according to Adensunkanni *et al*., and 16.5% according to Beniwal *et al*.[8,9,11,14]

We have compared the incidence of fecal fistula formation in primary closure group in which it was 7 (7.7%), consistent with other studies like that of Karmacharya and Sharma[16], while 1 (1.1%) in primary closure with omental patch group which is very low as compared with other series.

Incidences of mortality as reported by different studies are Beniwal *et al*., 10.5%; Purohit, 14.6%; Adensunkanni *et al*., 28%; Singh *et al*., 14.2%; and Talwar *et al*.., 16.4%.[4,6,9,11,14] Mortality in our study is very low mainly because of the selective group of patients. Group I patient mortality rate is 1 (1.1 %), while group II mortality rate is 3 (3.3%).

The incidence of other complications are almost same in both the groups like wound infection 33 (36.6%) in group I and 37 (41.1%) in group II, wound dehiscence in 6 (6.6 %) in group I and 7 (7.7%) in group II, intraabdominal abscesses in 5 (5.5%) in group I and 9 (10%) in group II.

## CONCLUSION

Laparotomy with primary closure is the mainstay of treatment in enteric perforation peritonitis depending on the bowel condition. Although the use of omentum is not new in surgery, its use in the ileal perforation is not very much stressed. Knowing its usefulness in other surgeries, we have used it in ileal perforation and found that omental patch technique is a reasonable option as compared with only primary closure. We recommend that it should be done in all cases of enteric perforation that fits the criteria of primary closure because of its obvious benefits. It is a very simple procedure and no extra time is required with less complication rates.
